# Incidence and predictors of acute kidney injury among women with severe pre-eclampsia at Mbarara Regional Referral Hospital

**DOI:** 10.1186/s12882-022-02972-8

**Published:** 2022-11-02

**Authors:** Mariam Hassan, Roland Mayanja, Wasswa G.M Ssalongo, Natumanya Robert, Lugobe Henry Mark, Okello Samson, Rose Muhindo

**Affiliations:** 1 Department of Obstetrics and Gynecology, Mbarara University of Science and Technology, Mbarara, Uganda; 2 Department of Obstetrics and Gynecology, Mbarara Regional Referral Hospital, Mbarara, Uganda; 3 Department of Medicine, Mbarara Regional Referral Hospital, Mbarara, Uganda; 4 Department of Internal Medicine, Mbarara University of Science and Technology, Mbarara, Uganda

**Keywords:** Severe pre-eclampsia, Eclampsia, Acute kidney injury

## Abstract

**Background:**

The presence of acute kidney injury (AKI) in pre-eclampsia complicates treatment including; increasing length of hospital stay and a need to access services like dialysis which are largely expensive in resource-limited settings. We aimed to determine incidence and predictors of acute kidney injury among women with severe pre-eclampsia at Mbarara Regional Referral Hospital in southwestern Uganda.

**Methods:**

We carried out a hospital-based prospective cohort study from 16 November  2018 to 18 April 2019, among pregnant women with severe pre-eclampsia followed up in the hospital. We enrolled 70 mothers with severe pre-eclampsia and eclampsia; we excluded patients with a history of chronic kidney disease, chronic hypertension, and gestational hypertension. Data on socio-demographics, laboratory parameters, health system, obstetric and medical factors were collected. Baseline serum creatinine, complete blood count, and CD4 T-cell count were all done at admission (0-hour). A second serum creatinine was done at 48-hours to determine the presence of AKI and AKI was defined as a relative change of serum creatinine value at least 1.5 times the baseline (i.e., at admission) within 48 h. The proportion of women diagnosed with acute kidney injury among the total number of women with severe pre-eclampsia was reported as incidence proportion. Univariate and multivariate logistic regression was used to establish the association between acute kidney injury and severe pre-eclampsia.

**Results:**

Incidence of acute kidney injury was high (42.86%) among women with severe pre-eclampsia. Antenatal care attendance was protective with an odds ratio of 0.14 (0.03, 0.73), p-value 0.020 at bivariate analysis but had no statistical significance at multivariate analysis. Eclampsia was an independent risk factor for acute kidney injury. (aOR 5.89 (1.51, 38.88), p-value 0.014.

**Conclusion:**

The incidence of acute kidney injury in patients with severe pre-eclampsia is high. Eclampsia is an independent risk factor of acute kidney injury. The findings of this study highlight the urgent need for more research and better perinatal care for these women.

## Introduction

Acute Kidney Injury (AKI) is defined as an abnormality of the excretory function of the kidney characterized by an accumulation of nitrogenous waste which includes urea and creatinine[1].

Incidence and factors associated with acute kidney injury in women with severe pre-eclampsia vary. Severe renal impairment occurs frequently when pre-eclampsia is complicated by abruption placentae or hemolysis, elevated liver enzymes, and low platelet count (HELLP) syndrome [2]. A history of pre-eclampsia in previous pregnancies increases the risk of end stage renal disease by 3 fold while this risk is increased to 5 fold if two or more previous pregnancies were complicated by pre-eclampsia[3].

The most common cause of AKI in pregnancy is pre-eclampsia, which is worsened with eclampsia, which can ultimately lead to an increase in maternal morbidity and mortality [4]. However in most women with pre-eclampsia, the glomerular filtration rate (GFR) decreases by no more than 30 to 40%, which results in small increases in the serum creatinine.[5]AKI requiring renal replacement therapy (RRT) is uncommon except in patients with very severe pre-eclampsia[5].

In Africa, AKI is a challenging problem because of the burden of disease, the late presentation of patients to health care facilities, and the lack of resources to support patients with established AKI in many countries[6]. A high proportion of AKI in low income countries occur in obstetric settings.

The presence of AKI in pre-eclampsia complicates the treatment including increasing the length of hospital stay, the need to access services like dialysis which are largely expensive. It is therefore paramount to know the incidence and predictors of AKI in the setting of Mbarara Regional Referral Hospital in Southwestern Uganda in mothers admitted with pre-eclampsia. Knowing the incidence and predictors of AKI in this population will help increase awareness among healthcare providers and will contribute to improving service delivery in our settings.

## Study design, setting and population

We prospectively enrolled70 participants on the maternity ward at Mbarara Regional Referral Hospital (MRRH) to determine the incidence and predictors of acute kidney injury in mothers admitted with severe pre-eclampsia. MRRH is a public regional referral hospital that serves as a teaching hospital for Mbarara University of Science and Technology. The hospital serves as a referral for the western region of Uganda but also receives patients from the neighbouring countries like Rwanda, Congo, and Burundi. The hospital offers specialized services, employs eleven obstetricians and 32 midwives together with resident doctors who perform over 10,000 deliveries annually. The hospital also can perform laboratory tests including chemistries, urinalysis, and ultrasonography among others. A resident nephrologist in the hospital can perform acute hemodialysis for patients who require the service.

The study population consisted of all women having severe pre-eclampsia and eclampsia between November 2018 and April 2019. We included all women who were diagnosed with severe pre-eclampsia/eclampsia defined as high blood pressure (≥ 140/90mmHg) plus one or more of the following symptoms of severity (headache, epigastric pain, blurring of vision, convulsions, and proteinuria plus 2 or 3 on urine dipstick). Women who had a history or documentation of chronic kidney disease, chronic hypertension and gestational hypertension were excluded. Eligible women were recruited prior to delivery until the sample size was attained. Consent was obtained and blood samples were taken for baseline renal function tests. Serum creatinine was measured using the kinetic colorimetric method (fixed rare) Jaffe reaction without deproteinization. After 48 h another sample was drawn for a 2nd creatinine. A relative change of serum creatinine value at least 1.5 times the baseline (i.e., at admission) within 48 h was considered AKI and patients were followed up by the study team while in hospital from the time of admission to when they were discharged. In this study, the KDIGO definition of AKI without urine criteria was used. Acute kidney injury was staged as follows: Non-AKI < 1.5 times change of the baseline creatinine, stage 1 as 1.5 to 1.9 times the baseline creatinine and stage 2 as 2.0 to 2.9 times the baseline and stage 3 which is defined as a change > 3.0 times the baseline creatinine. The definition of AKI in pregnancy has not been standardized. Different studies use different parameters (consensus definitions of AKI) which can range from a change in serum creatinine or the need for dialysis [7]. The physiological decrease in serum creatinine in pregnancy compared with similarly healthy, non-pregnant individuals is due to volume expansion and vasodilatation. This in turns leads to increased renal plasma flow and glomerular filtration rate resulting in an increased GFR [8]. The sample size was estimated based on the Fleiss formula (1981) for cohort studies.

The major outcome variable of the study was acute kidney injury.The primary exposure variable was severe pre-eclampsia while the secondary exposure variables were sociodemographic factors, healthcare system factors, obstetric factors which included gravidity/parity, gestation age at diagnosis of pre-eclampsia and admission to the hospital, postpartum hemorrhage, antepartum hemorrhage, eclampsia, HELLP syndrome and previous history of pre-eclampsia. The medical factors were a history of diabetes, and HIV infection.

### Statistical analysis

Data was captured using Redcap, and exported to Excel. All analyses were performed using Stata software Version 13.0. Continuous variables such as age were presented as means and standard deviation for normally distributed variables or median and interquartile range for variables not normally distributed. The incidence proportion of AKI was derived as the proportion of women diagnosed with acute kidney injury among the total number of women with severe pre-eclampsia/eclampsia. Bivariate and multivariate logistic regression models were used to identify predictors of acute kidney injury. Predictor variables were variables with odds ratio. Additional variables were included if on bivariate analysis they demonstrated a correlation with the outcome of interest (acute kidney injury) with a *P*-value < 0.5. Backward stepwise elimination was used to create the final model, and all variables with *P*-values < 0.05 in the final model were considered significant independent factors associated with acute kidney injury.

## Results

A total of 4547 obstetrics and gynecology admissions were conducted during the study period, out of which 75 met the inclusion criteria. Among those eligible, some participants refused to participate while others took their own discharge from the hospital before official discharge. 70 participants were recruited.

The mean age of our study participants was 27.5(± 6.4) years with 87.1% of them being married. 65.7% of the participants were housewives and 76.7% of the participants were referred from lower health facilities. A majority (85.3%) reported having attended antenatal care clinics. The commonest gestational age at delivery was 36 weeks and above. HELLP syndrome accounted for 27.1% of cases while 21.4% had eclampsia. The mean systolic blood pressure was 166 (± 28.5) while the diastolic was 108(± 16.8) mmHg. The mean baseline creatinine was 0.9 mg/dl (± 0.3). 68.6% of the participants had proteinuria and the mean hemoglobin level was 11.3 g/dl (± 2.4). The details of the participants’ baseline characteristics are shown in Table 1.

**Table 1:** Baseline characteristics of study participants

Characteristics	Overall N = 70 N (%)
**Age, in years, mean (SD)**	27.5(± 6.4)
**Education category**	
None	26 (37.1)
Primary	20 (28.6)
**Marital status**	
Married	61 (87.1)
Housewife	46 (65.7)
**Referring facility**	
HC III and IV	39 (76.5)
**Obstetric factors**	
Multigravida	51 (72.9)
ANC attendance, yes (N = 68)	58 (85.3)
**Gestational age at diagnosis**	
20–28 weeks	12 (17.1)
28–34 weeks	18 (25.7)
34–36 weeks	12 (17.1)
36 weeks & above	28 (40.0)
Abruption placenta	4 (5.7)
HELLP syndrome	19 (27.1)
Eclampsia	15 (21.4)
**Laboratory parameters**	
Haemoglobin in g/dl, mean (SD)	11.3 (± 2.4)
< 10 g/dl	13 (18.6)
> 10 g/dl	57 (81.4)
Proteinuria	48(68.6)
Baseline creatinine at admission in mg/dl, mean (SD)	0.90 (± 0.3)
HIV status, positive	5 (7.1)
SBP at admission in mmHg, mean (SD)	166 (± 28.5)
DBP at admission in mmHg, mean (SD)	108.8 (± 16.8)

Abbreviations: HC Health Center, ANC Antenatal Care, HIV Human Immunodeficiency Virus, HELLP- Hemolysis Elevated Liver Enzymes Low platelets, SBPSystolic Blood pressure, DBP Diastolic Blood pressure

### Incidence of acute kidney injury

**Fig. 1:**
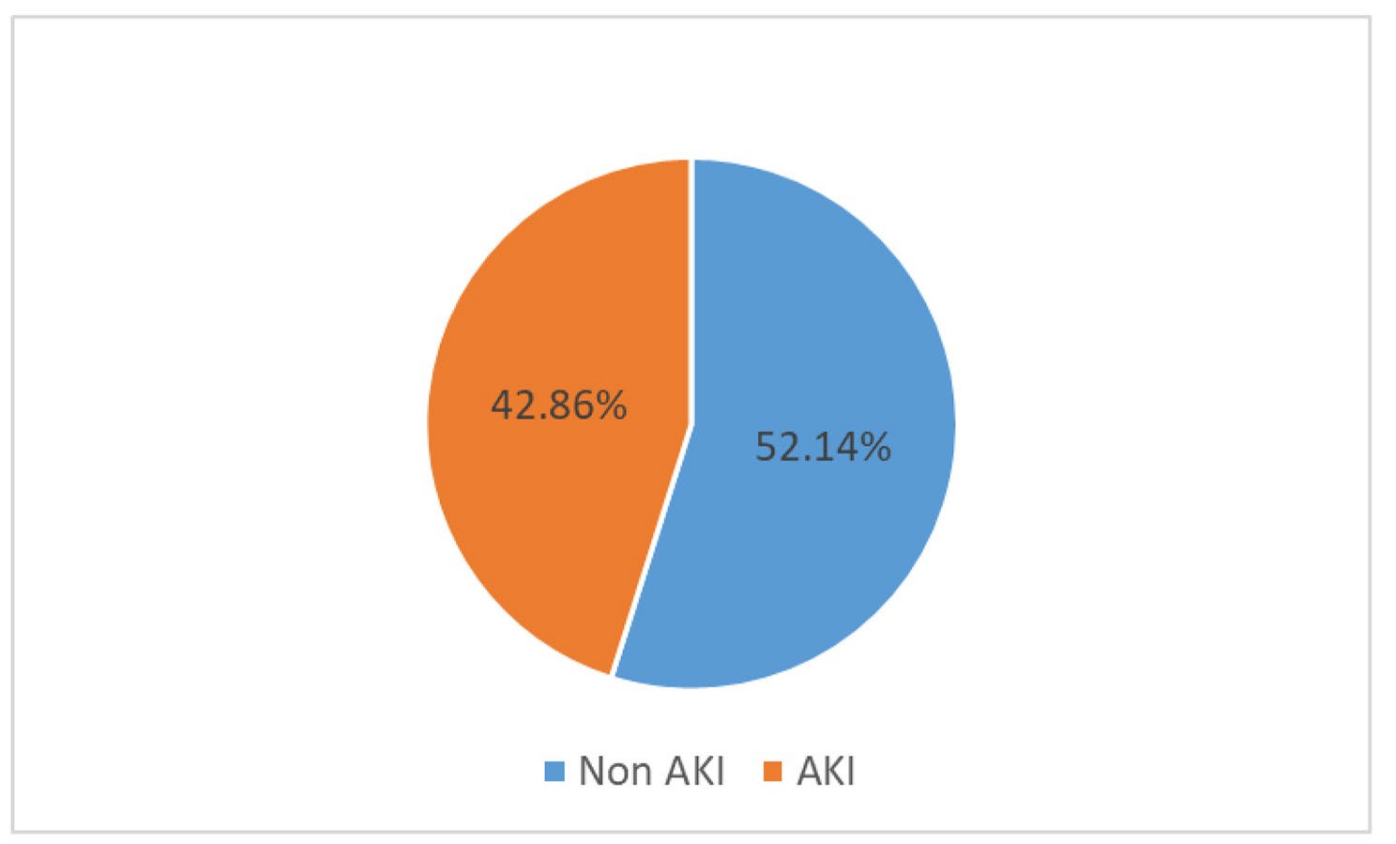
Incidence of acute kidney injury among mothers with pre-eclampsia

The incidence of acute kidney injury among women with severe pre-eclampsia/ eclampsia was 30/70 (42.86%) and is diagrammatically represented in Fig. 1.

**Fig. 2:**
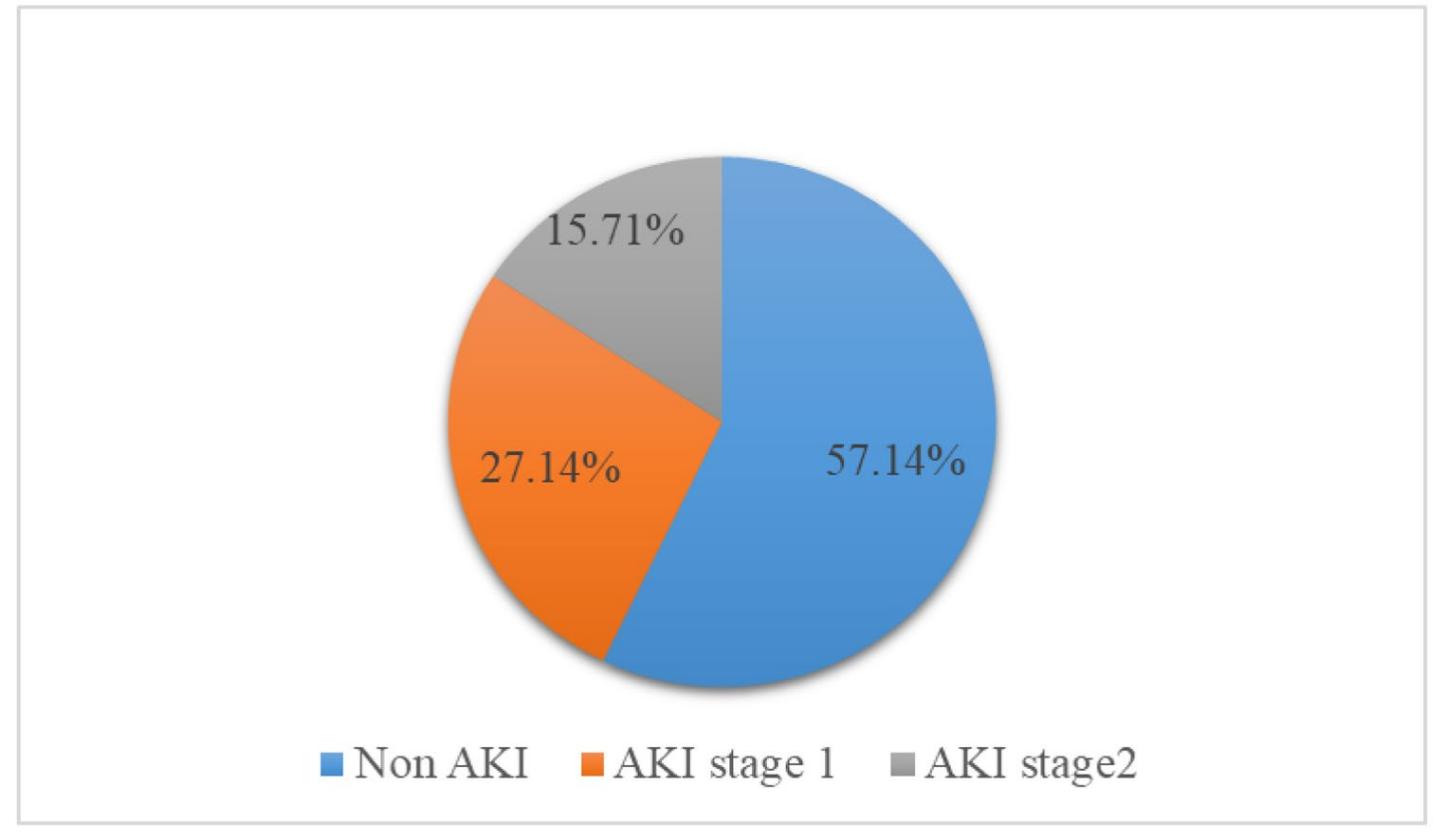
Chart showing stages of AKI among patients admitted with Pre-eclampsia

None of the study participants had severe AKI and neither did any of them progress to severe AKI or require hemodialysis as represented in Fig. 2. The stages of AKI in this study population are represented in Fig. 2.

Acute kidney injury was staged as follows: Non-AKI < 1.5 times change of the baseline creatinine, stage 1 as 1.5 to 1.9 times the baseline creatinine and stage 2 as 2.0 to 2.9 times the baseline. We had no patient in stage 3 which is defined as a change > 3.0 times the baseline creatinine.

### Predictors of acute kidney injury in logistic regression (both univariate multivariate analysis)

Antenatal care attendance was protective with a crude odds ratio of 0.14 (0.027, 0.73), p-value 0.020 in bivariate analysis but there was no statistical significance at multivariate analysis. Eclampsia was an independent risk factor of acute kidney injury (aOR 5.89 (1.51, 38.88), p-value 0.014. The details of the predictors of acute kidney injury among women with severe pre-eclampsia are shown in Table 2.

**Table 2:** Predictors of acute kidney injury by logistic regression

Characteristic	Bivariate analysis	p-value	Multivariate analysis	P-value
	**cOR** (95%CI)		**aOR (95%CI)**	
Age (years) 18–25 26–35 > 36	1 0.99(0.36–2.73) 0.52(0.11–2.39)	0.979 0.402		
ANC attendance				
No	Ref		Ref	
Yes	0.14 (0.027,0.73)	**0.020**	0.03 (0.0007,1.41)	0.075
Eclampsia				
No	Ref		Ref	
Yes	3.5 (1.04, 11.7)	**0.042**	5.89 (1.51, 38.88)	**0.014**
HIV status				
Negative	Ref		Ref	
Positive	2.18 (0.33, 14.09)	0.410	3.06 (0.28, 32.56)	0.35
SBP > 160 or DBP > 110				
No	Ref		Ref	
Yes	1.27 (0.58,5.62)	0.312	7.89 (0.81,76.5)	0.074
Gravidity				
2 or less	Ref			
3 or more	1.72 (0.65, 4.54)	0.268	2.32 (0.26,20.4)	0.44
Parity				
2 or less	Ref			
3 or more	1.71 (0.65, 4.46)	0.269	0.86 (0.96, 7.73)	0.89
Gestational age at delivery				
20–28	Ref			
28–34	1.4 (0.32,6.10)	0.654		
34 or more	0.93(0.25,3.46)	0.918		
Proteinuria				
No	Ref		Ref	
Yes	1.12 (0.40,3.12)	0.824	1.22 (0.28,5.20)	0.783
HELLP syndrome				
No	Ref		Ref	
Yes	2.31 (0.79, 6.77)	0.125	1.47(0.26, 8.05)	0.656
Previous pre-eclampsia				
No	Ref		Ref	
Yes	2.73 (0.72, 10.41)	0.139	4.55 (0.58, 35.66)	0.149
Abruptio placenta				
No	Ref		Ref	
Yes	1.17 (0.35, 3.95)	0.790	0.83 (0.003, 2.22)	0.138

Abbreviations: ANC Antenatal Care, HIV Human Immunodeficiency Virus, SBP Systolic Blood Pressure, DBP Diastolic Blood Pressure

## Discussion

In this prospective study, our main goal was to establish the incidence and predictors of AKI among mothers admitted with severe pre-eclampsia to the maternity ward of Mbarara Regional Referral Hospital (MRRH). We found a high cumulative incidence of AKI of 42.86% and eclampsia as the main independent predictor of AKI, within 48 h after admission.Our findings were lower than what was reported among severe pre-eclampsia/eclampsia patients in Cameroon among whom 66.7% developed acute kidney injury. This could be because a majority ( 72.5% ) of the enrolled participants in the study done in Cameroon had eclampsia [9] compared to our study which had only 21.4% patients with eclampsia. Furthermore, the Cameroon study was conducted in an intensive care unit and also had 7 years of follow-up. Data from developed countries suggests lower rates of AKI, with estimates as low as 1-2.8% [10]. However, in developing countries, the incidence of AKI is high ranging from 36–66.7% [11, 12], Cameroon (66.7% ) [13], Morocco (66.7%) [14]. The high incidence of AKI in developing countries may be attributed to the late presentation of patients to hospitals for care.

In our study, eclampsia was the main independent predictor of AKI (aOR 5.89 (1.51, 38.88), p-value 0.014. This is in agreement with a prospective hospital-based study done in India which revealed that acute kidney injury was common among patients with eclampsia as high as 25–50% [15]. This may be so because pre-eclampsia and eclampsia are associated with glomerular endotheliosis which decreases the glomerular filtration rate and renal blood flow. An increase in renal vascular resistance predisposes patients to AKI which is characterized by proteinuria and renal failure.

In our study, we noted that antenatal care attendance was associated with a reduction in the incidence of acute kidney injury compared to non-attendance; crude odds ratio 0.14, 95% C.I, 0.027, 0.73, p-value 0.02. However, this was not significant at multivariate analysis and there was no association between the number of times the mothers attended antenatal care and the incidence of AKI. There is evidence to suggest that women with severe pre-eclampsia who present with severe complications are likely to have attended ANC fewer times than those without major complications (Manandhar et al., 2013). This may be so because antenatal attendance is associated with early detection of disease and timely intervention due to the structured routine screening of blood pressure and urine protein during antenatal visits.

However,some studies found no association between the number of times of antenatal care attendance and the severity of pre-eclampsia on presentation [16, 17]. This may be because the disease progression pattern is most times unpredictable and acute complications can occur without warning symptoms.

The inability to use measure more sensitive renal biomarkers for the diagnosis of AKI was one of our study limitations. Serum creatinine takes time to rise and may lead to late detection of AKI. The timing of sample collection in this study was done at admission and 48 h later. This may have prohibited some cases being identified as it is possible that AKI might have been detected even after 48 h and within 7 days from admission. Furthermore, the physiological decrease in serum creatinine in pregnancy compared with similarly healthy, non-pregnant individuals which occurs due to volume expansion and vasodilatation which ultimately leads to increased renal plasma flow and glomerular filtration rate resulting in an increased GFR [8].

Urine output is not routinely monitored quantitatively unless in very sick or catheterized patients.

## Conclusion

The incidence of acute kidney injury in patients with pre-eclampsia/eclampsia is high (42.86%).

The presence of eclampsia in patients admitted with severe pre-eclampsia is associated with the development of AKI within 48 h after hospitalization. We recommend a larger prospective study that will include the use of biomarkers for acute kidney injury and closer monitoring of mothers with eclampsia for features of acute kidney injury. It is also important to increase both clinician and patient awareness to minimize the risk of AKI in women with pre-eclampsia.

## Data Availability

The datasets used during this study are available from the. corresponding author upon reasonable request.

## Abbreviations

AKI: Acute Kidney Injury
ALP: Alkaline phosphatase
AST: Aspartate aminotransferase
ALT: Alanine aminotransferase
ATN: Acute tubular necrosis
CBC: Complete blood cell count
CKD: Chronic kidney diseases
eGFR: Estimated Glomerular filtration rate
HELLP: Hemolysis, elevated liver enzymes and low platelet syndrome
LFT: Liver function test
MRRH: Mbarara Regional Referral Hospital
MUST: Mbarara University of science and Technology
OBS/GYN: Obstetrics and Gynecology
RFT: Renal function test
